# Fostering cultural responsiveness in physiotherapy: curricula survey of Australian and Aotearoa New Zealand physiotherapy programs

**DOI:** 10.1186/s12909-019-1766-9

**Published:** 2019-08-30

**Authors:** Maxine Te, Felicity Blackstock, Lucy Chipchase

**Affiliations:** School of Science and Health, Western Sydney University, Campbelltown Campus, Locked Bag 1797, Penrith, NSW 2751 Australia

**Keywords:** Curriculum, Cultural responsiveness, Entry-level education, Physiotherapy

## Abstract

**Background:**

Developing cultural responsiveness among physiotherapists is considered essential to promote quality and equity in healthcare provision for our culturally diverse populations. The aim of this study was to evaluate how entry-level physiotherapy programs in Australia and Aotearoa New Zealand (NZ) design curricula to foster the development of cultural responsiveness in physiotherapy students. Further, the challenges of integrating educational content and approaches, and the perceptions of the effectiveness of these curricula were also explored.

**Methods:**

A cross-sectional telephone survey with closed and open-ended questions, was conducted with 18 participants representing 24 entry-level physiotherapy programs (82% of all programs) in Australia and NZ between May and September 2017. Data were analysed descriptively in the form of frequencies, percentages or ratios as appropriate. Open-ended responses were thematically analysed.

**Results:**

Results suggest variability in the structure, and teaching and assessment methods used across all programs. The majority of programs appeared to rely on didactic teaching methods, along with knowledge based and implicit assessment methods. The main challenges reported were that cultural responsiveness was thought to be perceived by academic staff as unimportant and that the curriculum was perceived to be ‘overcrowded’. Participants also felt there was room for improvement despite perceiving the curriculum to be effective at fostering cultural responsiveness.

**Conclusion:**

Results provide insight into the educational content and approaches integrated in entry-level physiotherapy curricula in Australia and NZ, and suggest opportunities for further research and development to foster cultural responsiveness among physiotherapy students.

**Electronic supplementary material:**

The online version of this article (10.1186/s12909-019-1766-9) contains supplementary material, which is available to authorized users.

## Background

The development of cultural responsiveness among physiotherapists is considered to be an essential strategy to enhance the quality of healthcare, and improve the health outcomes of indigenous and culturally and linguistically diverse (CALD) populations [1–4]. At an individual level, cultural responsiveness refers to the ability of healthcare professionals to deliver care that is safe, respectful and responsive to the health beliefs and practices, and the cultural and linguistic needs of their patients [2, 5, 6]. While the development of cultural responsiveness is a lifelong journey, entry-level physiotherapy programs should design curricula to include educational content and approaches that foster the development of cultural responsiveness in physiotherapy students [1, 7, 8].

The “Physiotherapy Practice Thresholds in Australia and Aotearoa New Zealand” explicitly state that cultural responsiveness is an ‘essential component’ of physiotherapy practice [9]. The ‘essential components’ are a list of behaviours that apply to all key competencies outlined in the Physiotherapy Practice Thresholds. As such, physiotherapists in Australia and Aotearoa New Zealand (NZ) should always “*consider each client as a whole, adopt client-centred and family/whānau focused (where relevant) approaches and prioritise cultural safety and cultural respect*” [9]. This commitment to culturally responsive practice has been driven by government health policies and legislations that aim to reduce the health disparities experienced by Māori, Aboriginal and Torres Strait Islander peoples and people from CALD communities. In NZ, the founding document, Te Tiriti o Waitangi or the Treaty of Waitangi, underpins all legislation, policy, and practice to improve health outcomes for Māori people [10]. Additionally, the *Health Practitioners Competence Assurance Act 2003* (NZ) requires that health regulatory authorities ensure registered health professionals are culturally responsive in their practices [11]. In Australia, government policies, such as Closing the Gap and Australia’s Multicultural Policy, aim to improve access, equity and health outcomes for people from Aboriginal and Torres Strait Islander and CALD communities, respectively [12, 13]. To meet the boards’ requirements, entry-level physiotherapy programs must include learning outcomes and assessment of the attainment of those outcomes, to ensure that their graduates can work safely, respectfully and autonomously in culturally diverse societies.

Published research exploring the development and exhibition of cultural responsiveness in physiotherapy is sparse. There is some evidence that suggests physiotherapists may not be culturally responsive. For example, a qualitative study by Lee et al. [14] reported that Australian physiotherapists had negative attitudes towards people from CALD communities, and tended to stereotype ethnocultural groups. Australian physiotherapists were also reported to hold negative perceptions about using interpreters and had misconceptions about communication with people with limited proficiency in English [15, 16]. These issues are recognised as factors that lead to culturally inappropriate care and have been identified as important areas that should be addressed by health professional education programs, such as physiotherapy [17–20].

To date, only one published study has explored the inclusion of educational content and approaches related to culture or cultural responsiveness in entry-level physiotherapy programs [21]. This study was conducted in one state of the United States, and captured the status of the physiotherapy curricula 20 years ago, limiting the generalisability of results to current day teaching practices internationally [21]. Other studies evaluating educational content and approaches related to culture or cultural responsiveness in the curricula have been conducted in medicine, nursing or dentistry [22–34]. This work, conducted mainly in the United States, demonstrated that educational content and approaches related to culture or cultural responsiveness were integrated into most curricula, with wide variation existing in content, teaching and assessment methods, and level of integration. Overall, these studies provide an understanding of how and whether health professional education programs appropriately design curricula to foster cultural responsiveness.

With little published about physiotherapy curricula, how and whether physiotherapy students are appropriately supported in developing cultural responsiveness is not known. Therefore, the purpose of this study was to evaluate how entry-level physiotherapy programs in Australia and NZ design curricula to foster the development of cultural responsiveness in physiotherapy students. This is particularly important given the indigenous populations and the increasing culturally diverse population in Australia and NZ [35, 36].

## Methods

### Study design

A descriptive, cross-sectional exploratory design was used. A semi-structured telephone interview, with closed and open-ended questions, was used for data collection. Telephone interviews were chosen as they have been demonstrated to have lower rates of missing responses compared to postal surveys [37, 38]. Ethics approval was granted from the University Human Research Ethics committee (Approval No: H11909).

### Design of the Interview Guide

A focus group was used to facilitate the development of the interview guide and to ensure content validity [39–41]. The focus group provided a method to identify relevant and appropriate concepts to generate interview questions [42]. Participants who had experience teaching material related to cultural responsiveness, and/or had experience in teaching or curriculum development in entry-level health profession programs in the tertiary education sector were invited to participate. Participants were recruited via email. Five academic staff from Western Sydney University consented to participate. Two participants were from health science with one specialising in education related to cultural competency. The other three participants were from health and physical education, paramedicine and physiotherapy.

The principal researcher (MT) facilitated the focus group. Questions asked during the focus group were designed to facilitate an in-depth exploration of the concepts, educational content, and approaches related to cultural responsiveness that would contribute to the generation of the interview questions. Relevant key elements such as educational approaches, assessment methods and content areas perceived important for developing cultural responsiveness, were also extracted from a literature search of survey studies investigating cultural responsiveness in other health profession programs to help guide the focus group discussion and question development. For example, the focus group was asked to describe how they integrated content related to culture or cultural responsiveness into their teaching or asked to list content areas that they considered important.

The data from the focus group was audio-recorded. The principal researcher (MT) transcribed, and analysed the data using an inductive approach to identify themes [43]. After the themes and concepts were identified, a draft interview guide was developed. Questions in the draft interview guide were also modelled using previous surveys of curricula identified in the literature [22, 26–29, 31]. The initial draft of the interview guide was reviewed by the research team to ensure appropriate wording, spelling, format sequencing, and that the questions were relevant to the aims of the study. Slight changes to wording were made to enhance the clarity and comprehensibility of the questions. Due to the small participant response pool, the interview guide was pilot tested with an academic from the physiotherapy department at the lead institution to ensure clarity, correct interpretation of questions, and assess completion time. The final interview guide contained nine structured, closed-ended questions about the integration of content related to culture or cultural responsiveness in the curriculum, and two open-ended questions. The first open-ended question was about the perceived challenges to integrating educational content and approaches related to culture or cultural responsiveness, and the second about their perceptions of the effectiveness of the curricula in fostering cultural responsiveness (see Additional file 1 for the final interview guide).

### Data collection

Twenty-nine physiotherapy programs in 21 universities were identified from the listings of accredited programs of study found on the Physiotherapy Board of Australia, and the Physiotherapy Board of New Zealand websites, accessed at the beginning of 2017 (http://www.physiotherapyboard.gov.au; https://www.physioboard.org.nz). Every university that enrolled students in an entry-level physiotherapy degree in Australia or New Zealand was invited to participate. A letter of invitation was emailed to the discipline lead for physiotherapy. If the discipline lead agreed to participate but was not the most appropriate person to complete the interview, he or she was requested to nominate another academic staff member in their school/department. There were no eligibility criteria for participating in this study due to the variability of staff roles and/or responsibilities across different programs. As such, programs may or may not have specific staff in the department involved in curriculum development for fostering cultural responsiveness. A letter of invitation was then sent via email to this academic staff. Informed and written consent was obtained from the discipline lead or nominated academic staff prior to data collection. A copy of a blank interview guide was also emailed to each participant before the interview to allow them time to collect any relevant material (Additional file 1). Participants were provided with flexibility in how they collected the information. Generally, participants were expected to gather information from academic staff responsible for teaching into the physiotherapy program or refer to learning guides on subjects offered in the course. All telephone interviews were audio-taped and transcribed verbatim by the principal researcher (MT). The written transcripts were cross checked against each audiotape for accuracy.

### Analysis

Closed-ended questions with ordinal or nominal responses were entered and coded in numerical format on a Microsoft Excel spreadsheet. Data were analysed descriptively in the form of frequencies, percentages or ratios as appropriate.

Open-ended responses were analysed inductively using qualitative thematic analysis [43]. First, transcribed data was read and re-read to allow familiarisation of the data, and any comments were written down during this phase. The initial coding of the transcript was carried out by the principal researcher (MT). All coded extracts were printed and cut out, and organised into meaningful groups. Coded extracts were then refined and categorised into themes. A codebook was then developed [44, 45]. One research team member (LC) who was not involved in data collection and the initial stages of data analysis independently coded 20% of the data using the codebook. Discrepancies related to the coding structure were discussed, codes were added, combined or deleted and the codebook was then revised [46, 47]. The data were independently coded again and results compared. This process was repeated until all inconsistencies were addressed and an agreement was reached on all coded data. The principal researcher (MT) then recoded all the data independently. Memo writing of the coding and analysis processes was performed throughout [48]. Member checking of the themes was also conducted to ensure the validity of the findings and interpretations [49]. All participants were provided with a written summary of the themes to review and were asked to confirm whether the themes represented their perceptions [50]. Two of the 18 participants responded, and no changes were required.

## Results

Eighteen universities agreed to participate in the study (86% response rate) and data was collected for 24 entry-level physiotherapy programs (82% of all physiotherapy programs in Australia and NZ). Demographic data for the types of programs and the number of programs that participated in this study are outlined in Table 1.

**Table 1:** Demographics of entry-level physiotherapy programs in Australia and Aotearoa New Zealand

Program Type	Duration of Programs	Postgraduate Program^a^	Number of Program Types in this Study^b^
Bachelors	4 years	No	14 (88%)
Bachelors/Masters (double degree)	4 or 5 years^c^	No	2 (100%)
Graduate Entry Masters	2 years	Yes	6 (75%)
Extended Masters	2 or 3 years^c^	Yes	2 (67%)

^a^Requires a bachelor degree qualification in a related health or medical science area to enrol into the program

^b^Percentages (%) represent the response rate for each type of program offered in Australia and NZ at the time of data collection

^c^Year variations are due to differences in university course structures. For example, semesters or trimesters

### Program structure

In two out of 24 entry-level programs, content related to culture or cultural responsiveness was integrated into the curriculum as a stand-alone subject, while 15 programs reported content to be integrated across the curriculum in a number of subjects, and seven programs had a mixture of both. The term ‘subject’ in this study refers to semester-length courses or units. Stand-alone subjects were delivered in the first half the curriculum of the programs (i.e. first two years of the bachelor or bachelor/masters programs, or first year of the graduate entry masters (GEM) programs). Five out of nine stand-alone subjects focused on Indigenous or First Peoples’ health and issues. The other four stand-alone subjects were focused on social determinants and/or communication.

Overall, there were 165 different subjects identified that addressed or embedded content related to culture or cultural responsiveness. Table 2 outlines the percentage of subjects identified across the different year levels for each program type. Identified subjects were also categorised broadly as a ‘stand-alone indigenous health’ subject, a ‘core physiotherapy’ subject such as cardiorespiratory, neurological or musculoskeletal physiotherapy practice, a ‘social determinants and health’ based subject, a ‘professional practice and communication’ based subject, a ‘community care and/or complex cases’ subject or a ‘clinical placement’ subject. Table 3 demonstrates the percentage of different types of subjects that were identified based on the six broad categories.

**Table 2:** Percentage of subjects across different year levels for each program type

	Year level				
Program type	1st year	2nd year	3rd year	4th year	5th year
Bachelors	24	19	16	38	x
Bachelors/Masters	21	14	1	36	21
Graduate Entry Masters	40	60	x	x	x
Extended Masters	46	29	7	x	x

**Table 3:** Percentage of different types of subjects identified

Type of subject	Percentage (%)
Stand-alone indigenous health	3
Social determinants and health	7
Community care and/or complex cases	8
Professional practice and communication	16
Core physiotherapy	32
Clinical placement	32

### Content areas included in the curricula

**Fig. 1:**
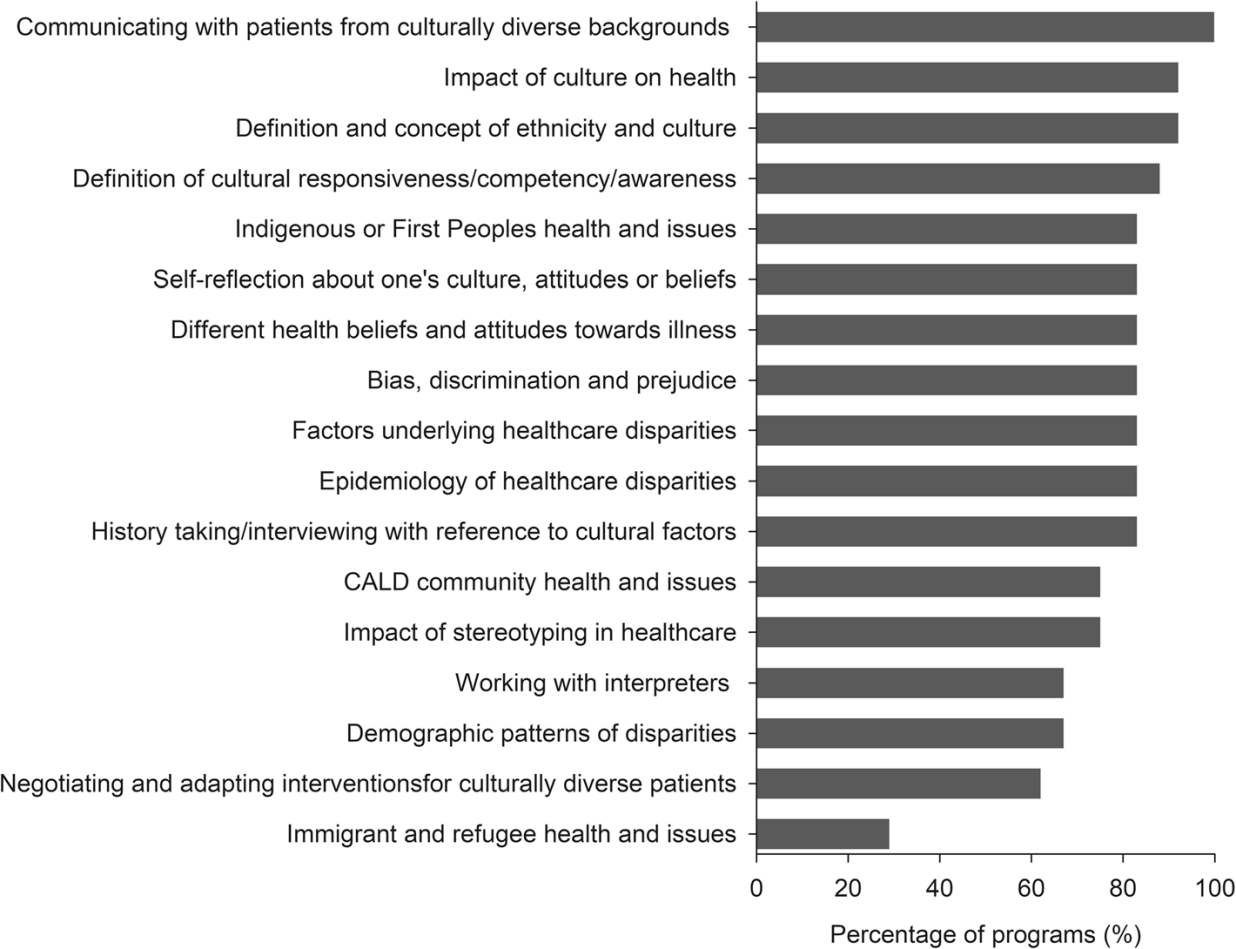
Percentage of programs that included the content areas related to culture or cultural responsiveness

Figure 1 demonstrates the percentage of programs that included each of the content areas related to culture or cultural responsiveness in the curricula. All programs included material about communicating with patients from culturally diverse backgrounds, most programs covered content related to indigenous (Māori in NZ or Aboriginal and Torres Strait Islander in Australia) populations, and concepts or definitions of culture, ethnicity or cultural responsiveness, while fewer programs (25%) covered immigrant and refugee health and issues.

### Teaching methods used to deliver content related to cultural responsiveness

**Fig. 2:**
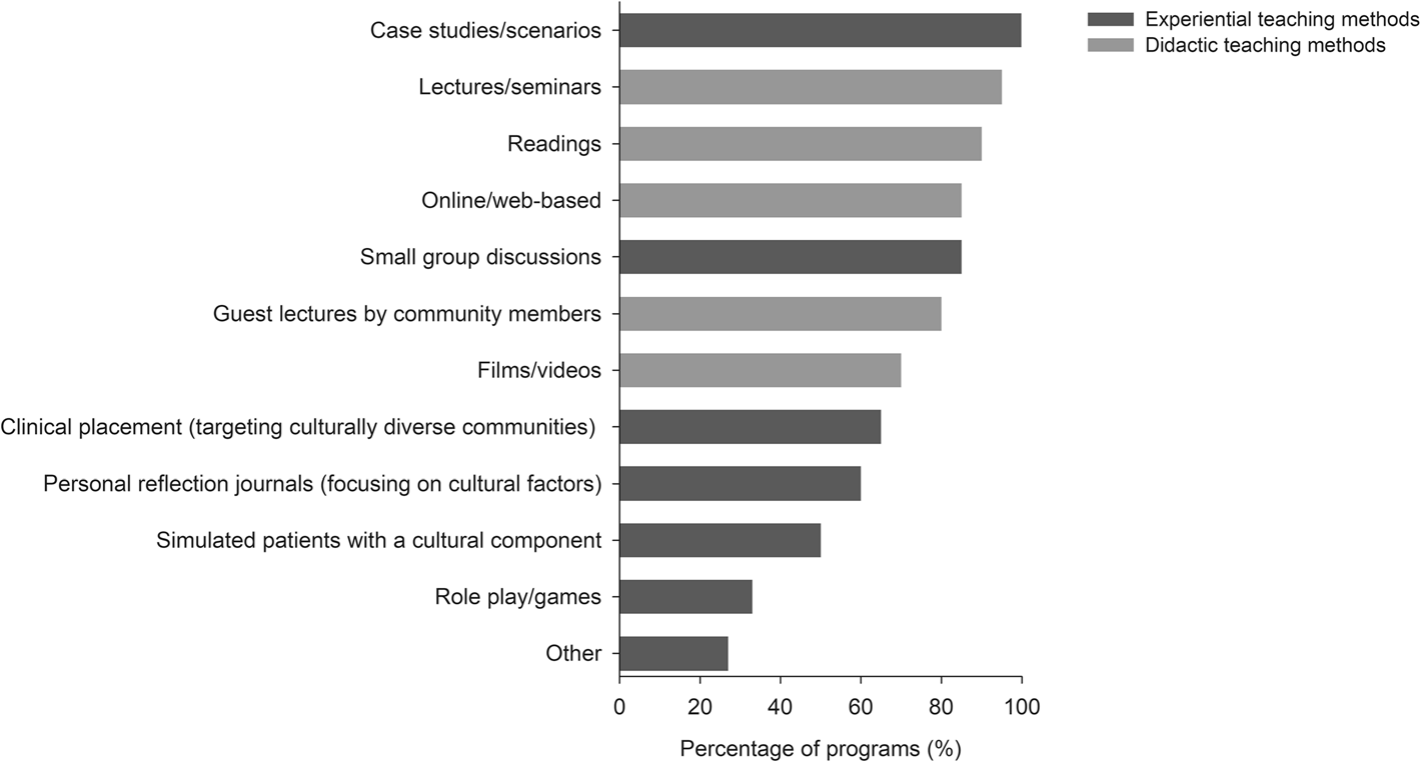
Percentage of programs that used each of the teaching methods to support the development of cultural responsiveness

Figure 2 demonstrates the percentage of programs that used each of the teaching methods to deliver content related to culture or cultural responsiveness. The most frequently reported educational methods were case studies or scenarios (100%), lectures/seminars (95%), small group discussions (85%), online/web-based (85%), and readings (90%), while fewer programs used methods such as role plays (33%) and simulations (50%). Other educational methods included leadership debates, volunteering with local communities, field trips to the local indigenous community areas or cultural centres, and overseas study abroad programs. Overall, most programs appeared to use didactic teaching methods, such as lectures, online and films/videos, compared to experiential teaching methods, such as simulation-based learning and immersion in culturally diverse healthcare communities.

### Assessment methods used to assess learning outcomes related to cultural responsiveness

**Fig. 3:**
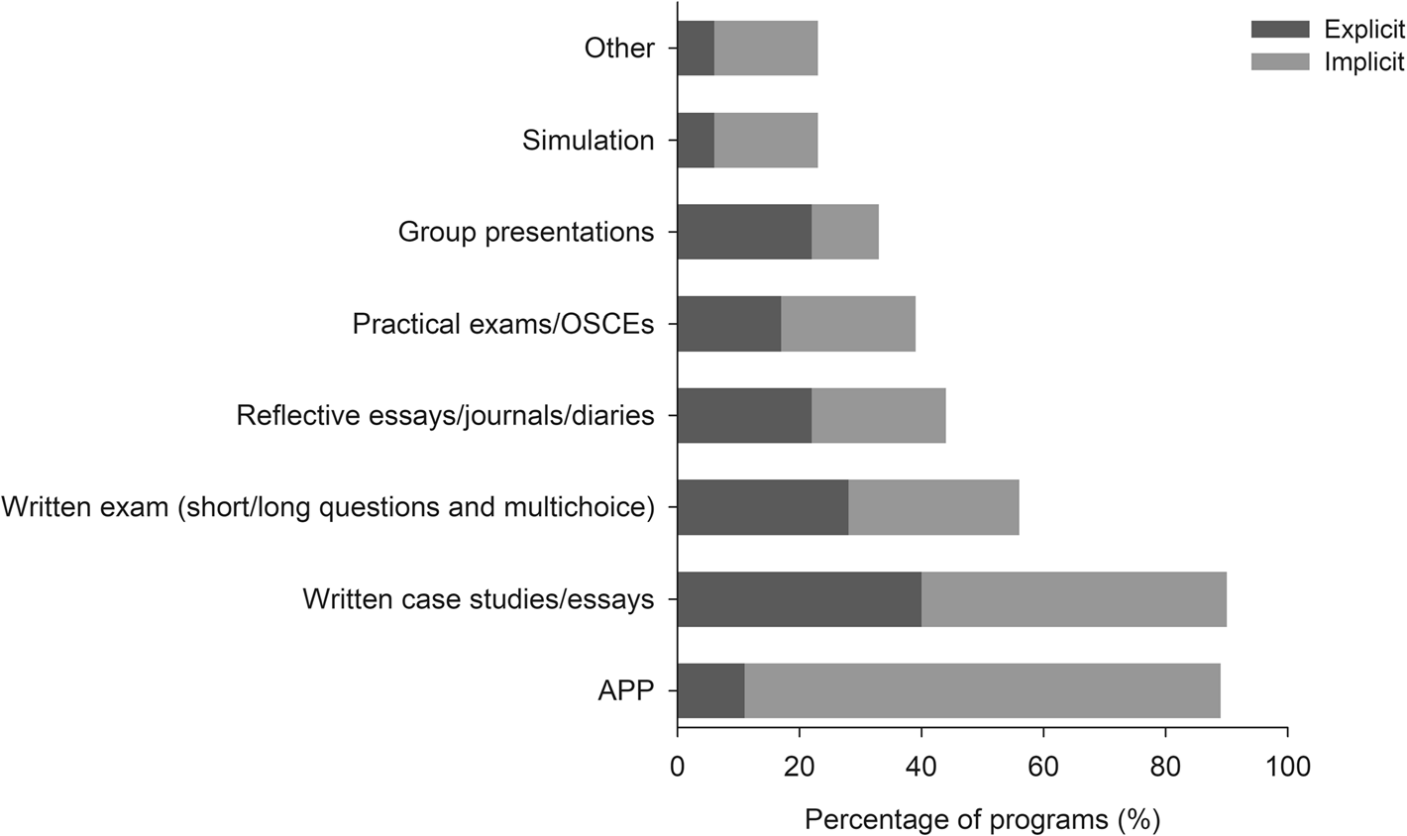
Percentage of programs that used each of the types of assessment methods to support the development of cultural responsiveness

Figure 3 illustrates the percentage of programs that used each of the assessment methods to assess learning outcomes related to culture or cultural responsiveness. An assessment method was considered explicit if the assessment criteria focused on learning outcomes related to culture or cultural responsiveness. The assessment method was considered implicit if the evaluation of student learning in reference to culture or cultural responsiveness was implied; the assessment criteria focused on learning outcomes not directly related to culture or cultural responsiveness, but potentially influenced by a student’s cultural responsiveness. For example, an explicit assessment could be a practical exam evaluating whether cultural factors were considered appropriately during history taking, while an implicit assessment could be a practical exam assessing broad skills such as communication skills and professionalism.

The most frequently reported methods of assessment were written case studies/essays (89%), the use of the Assessment of Physiotherapy Practice (APP), a standardised evaluation tool used by clinical educators in Australia and NZ to assess workplace based performance of entry-level physiotherapy students (89%) [51], and written exams (short/long questions and multiple choice) (56%). Fewer than half of the programs used practical exams (28%), simulations (22%), and reflection journals (44%) to assess the development of cultural responsiveness. Other types of assessment methods included student led seminars, journal clubs, debates, or poster presentations. Overall, a greater proportion of implicit assessments were used compared to explicit assessments.

### Perceived value of resources that inform content and teaching

**Fig. 4:**
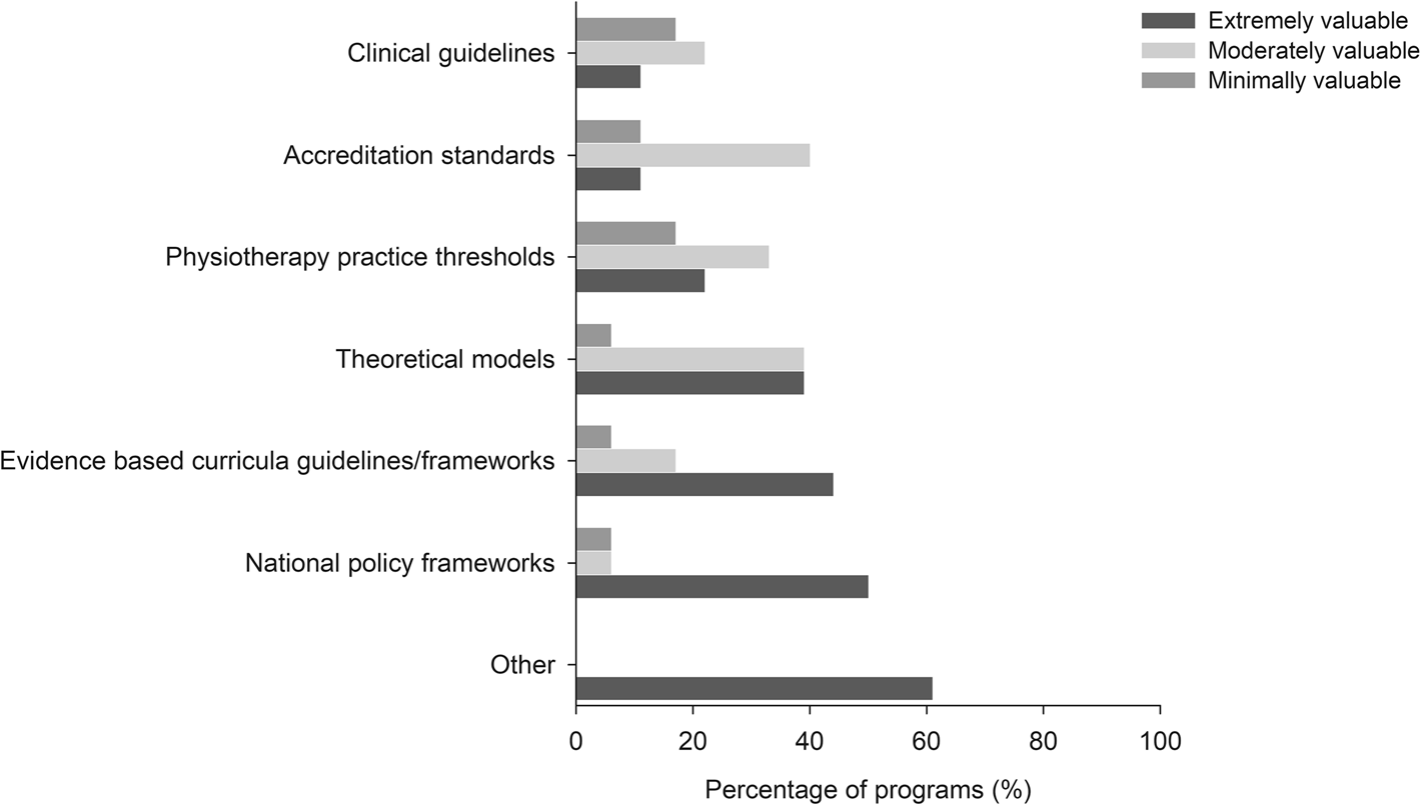
Percentage of programs that used each of the resources to inform content and teaching of cultural responsiveness in the curricula

Figure 4 outlines the percentage of programs that used each of the resources to inform the content and teaching that supports the development of cultural responsiveness and the perceived value of the used resources. The most frequently used resources were theoretical models (89%), and the Physiotherapy Practice Thresholds (83%). However, national policy frameworks (50%), and evidence-based curricula guidelines (44%) were perceived by participants to be the most valuable in informing content and teaching in the programs. Other resources considered extremely valuable by participants included personal experience and expertise from teaching staff who were of a Māori or Aboriginal and Torres Strait Islander background, consultations with local community groups and people working in culturally diverse areas, consultations with the Indigenous or First Peoples engagement unit/liaison from the University, and the University reconciliation action plan for Aboriginal and Torres Strait Islander populations.

### Open-ended questions

Six main themes were identified from the data which represented participants’ perceived challenges integrating educational content and approaches to foster cultural responsiveness. One prominent theme was identified from the data which represented participants’ perceptions about the effectiveness of the curriculum to foster cultural responsiveness. These themes are outlined below. Quotes supporting each theme are displayed in Tables 4 and 5.

**Table 4:** Summary of themes – Challenges in integrating content related to culture or cultural responsiveness

Theme	Sub theme	Examples
Perceptions of unimportance	Lack of understanding, awareness and/or interest	“I suppose a challenge is the staff designing the program being aware of cultural responsiveness and understanding that and prioritising that in a way they are including it not just in their teaching but their assessment … I suppose it’s an understanding of the educators that’s the first thing” [P3]
	Teaching is focused on areas perceived to be important to physiotherapy	“I think there’s a lot of drive to include clinical or biomedical content, I guess non patient-centred aspects in the curriculum, and I guess that comes from the history of physiotherapy and the focus on the body … traditionally there’s been less attention of peoples’ opinions and experience of health, and on population health and social justice …” [P5]
Overcrowded curriculum		“The next challenge is then sort of getting through the processes and trying to embed it within our programs without it being at the expense of other things we need …” [P11]
Difficulty with access and use of resources	Difficult to access and/or find appropriate teaching staff	“I guess a challenge would be finding lecturers with appropriate skills and expertise in the area” [P18]
	Difficult with finding and/or using resources	“… there is a lot of demographic or sociological kind of studies about how cultural groups behave or respond but really there hasn’t been a lot of intervention kind of research or trials to look at - if we do this, how does that improve cultural responsiveness? Therefore, it’s almost impossible to teach students how to behave [in a] culturally responsive manner.” [P1]
Challenging to teach		“… internationally there’s very few examples of very strong culturally responsive practice to draw on so it’s some way easy to teach around things like health and equity that you can usually demonstrate examples, but to think through and imagine, and create ways of working that are different but are useful to diverse communities that involve sort of flexibility and attentiveness and a change in clinician behaviour and attitude, I think that probably the teaching staff struggle with that” [P6]
Ensuring appropriate integration		“… I think that a real challenge is working out how to cover it appropriately and where” [P8]
Students’ perception of irrelevance		“I think there’s also the perception of students as well, unfortunately they don’t see the relevance … that’s the case with a number of physiotherapy modalities and techniques, they don’t see the relevance to the social side of things...” [P17]

**Table 5:** Summary of themes – Perceived effectiveness of curriculum

Themes	Sub themes	Examples
Perceptions of effectiveness	Ability of program to facilitate understanding and awareness	“I think we do … I think our health and wellbeing collaboration is really good at that in terms of exposing our students to a wide range of things, of different groups and trying to get them to really look at healthcare from that person’s perspective …” [P12]
	Effective, although room for improvement	“Yes … but I think that we can teach students how to adapt their interventions for people, and that’s where I don’t think we’ve made that link yet. So how have you changed your management approach of what you actually do to get better patient outcomes, better engagement, better retention in people from culturally diverse backgrounds? So, I think that’s what we can do better …” [P15]

### Challenges with integrating educational content and approaches

#### Perceptions of unimportance

Participants felt that academic staff who perceived cultural responsiveness as unimportant were less likely to focus their teaching on content related to culture or cultural responsiveness. Perceptions of unimportance were related to a lack of understanding, awareness, or interest by academic staff about cultural responsiveness, and because the conventional focus of teaching was based on other topics perceived to be important in physiotherapy.

#### Over-crowded curriculum

The limited amount of time available in the curriculum for students to develop competence across the breadth of physiotherapy was perceived to be a significant barrier. Participants found it challenging to find time to include content related to culture or cultural responsiveness, as the curriculum was already crowded with the content required for accreditation and competence in physiotherapy practice.

#### Difficulties with accessing and using resources

Participants found it difficult to access and find appropriate staff [with expertise] to teach content related to culture or cultural responsiveness. In particular, staff of Māori, Aboriginal and Torres Strait Islander or CALD backgrounds were perceived to have expertise. Participants also found it difficult to find relevant resources or use available resources to foster cultural responsiveness.

#### Challenging to teach

Participants found it challenging to teach content related to culture or cultural responsiveness. Participants noted it was challenging to ensure teaching did not stereotype ethnocultural groups and was not tokenistic, covered the breadth of content for many different ethnocultures, and facilitated the development of attitudes and behaviours required to be culturally responsive.

#### Ensuring appropriate integration

Ensuring that material was integrated appropriately in the curriculum was perceived to be a challenge. This was described as being related to uncertainty about how and where to integrate content to best facilitate cultural responsiveness.

#### Students’ perceptions of irrelevance

Some participants reported that it was a challenge to instil the importance of cultural responsiveness as students were thought to see culture and culturally responsive practice in physiotherapy as irrelevant or not important.

### Perceived effectiveness of the curriculum

Participants perceived the curriculum to be effective in fostering cultural responsiveness. Participants felt that the curriculum appeared to facilitate an understanding and awareness of the importance of culture in health among students. However, participants also perceived that there was room for improvement. Potential improvements included teaching students to integrate cultural knowledge into practice and adapt interventions, teaching processes to obtain cultural information, using appropriate assessment methods, and having a greater focus on CALD communities, health promotion and advocacy, and on patient-centred physiotherapy.

## Discussion

This is the first study to describe how entry-level physiotherapy programs across Australia and NZ design curricula to foster cultural responsiveness in physiotherapy students. Viewed together, all programs were integrating educational content and approaches related to culture or cultural responsiveness. This is encouraging given that health professionals have a legal and moral obligation to be culturally responsive in their practice and that education during the pre-professional years is considered one strategy to tackle the health inequities and to ensure quality healthcare for indigenous and CALD communities [1]. However, there was variability in the structure, teaching and assessment methods used, and the types of resources used to inform teaching. The majority of programs appeared to rely on didactic teaching methods, along with knowledge based and implicit assessment methods. Additionally, the main challenges reported were that cultural responsiveness was thought to be perceived by academic staff as unimportant and that the curriculum was perceived to be already “overcrowded”. Participants also felt there was room for improvement despite perceiving the curriculum to be effective in fostering cultural responsiveness. These findings highlight areas for improvement and should be considered if the profession is to ensure graduates are equipped with the knowledge, skills and the moral foundation to meet the healthcare needs of indigenous and CALD communities.

The variability in curricula across programs potentially reflects the accreditation standards and thresholds statements that programs must meet to ensure that their graduates are eligible for registration in Australia and NZ [9]. The accreditation process, using these thresholds and standards, is broad and open, allowing for variability and flexibility in curricula design. Consequently, content areas, and teaching and assessment methods are likely influenced by the local context or personal factors [52–54]. For example, universities located in culturally diverse communities may have a larger focus on cultural factors, or academic staff who have a personal interest in culture or cultural responsiveness may have a greater focus in their teaching. Additionally, the different socio-political dynamics and policies, particularly related to the indigenous communities are likely to have influenced the curricula.

The majority of programs appeared to rely on didactic teaching methods rather than methods facilitating experiential learning. Didactic teaching methods involve instruction and information to the learner, where the learner passively obtains knowledge [55]. Methods that support experiential learning involve facilitating learning through actively engaging the learner in direct experiences, and allowing them to learn through ‘doing’ and ‘reflecting’ [56]. Although the finding of reliance on didactic teaching methods was based on the authors’ classification, experiential learning methods such as role plays, simulation and clinical placements with a cultural focus, that provide direct experiences or participatory hands-on activities appeared to be less utilised. The theoretical and conceptual underpinnings of culturally responsive healthcare practice highlight that development occurs through human interaction and involves a process of critical reflection and action [57, 58]. This is because cultural responsiveness involves complex skills such as patient-centred communication and problem solving, and also requires the development of moral reasoning, open-mindedness and a critical consciousness on current practices and the healthcare system [57–59]. For this reason, experiential learning involving practical or authentic situations are recommended to encourage students to better understand the individuals they may work with, and allow them to practice, reflect on their experiences and see the effects of their actions, and also receive critical feedback [58]. While there is some research demonstrating the successful application and potential benefits of experiential learning approaches, such as community or international service learning in physiotherapy and other health disciplines, these studies do not explore the impact on patient outcomes and ongoing practice beyond graduation [60–63]. Therefore, research exploring effective teaching methods that lead to improved patient outcomes is required to ensure students learn to effectively support the health of culturally diverse communities.

Cultural responsiveness in students was assessed by most programs using knowledge based assessment methods, compared to practical, reflective or performance based assessment methods. Knowledge based assessments are usually written assessments that test factual recall and applied knowledge. These assessments provide little evidence of actual performance, skill or behaviour, which are considered key components of cultural responsiveness [2, 3, 64]. In contrast, performance based assessments are usually practical tests that assess knowledge, and evaluate actual performance, skill or behaviours [55, 65]. In this study, written case studies/essays and written exams were used in greater proportion to performance based assessments such as practical exams or simulation. The limited use of performance based assessments are also evident in the wider healthcare literature. As such, a majority of studies evaluating the impact of interventions on the development of cultural responsiveness use self-reported questionnaires, which assess knowledge or perceptions. To evaluate the complex skills, and behaviours associated with developing cultural responsiveness, performance based assessments are recommended to assess the ability of students to apply knowledge into practice [66]. However, research is first needed to understand the features of performance based assessments that would provide greater confidence that students have advanced in their development of cultural responsiveness.

How learning outcomes are assessed also influences learning, and assessment of cultural responsiveness in physiotherapy curricula appear to be done implicitly. Implicit assessments obscure the specific learning outcome that students need to successfully learn and then demonstrate. Implicit assessments may also send an unintended message that demonstrating good physiotherapy skills, in general, would result in carryover to performance in contexts with people from indigenous or CALD communities. As such, students may overlook their development of cultural responsiveness, or not demonstrate culturally responsive skills in the implicit assessment task. Despite these concerns, implicit assessments should not be completely disregarded as a process in assessing cultural responsiveness. Many skills in physiotherapy, such as communication and interpersonal skills, are influenced by cultural responsiveness and are difficult to explicitly separate [67]. Therefore, to adequately assess cultural responsiveness, educators need to ensure that the components and nuances of culture are considered during assessments, and that students are challenged in a cultural context and know they need to learn and demonstrate cultural responsiveness in the broad spectrum of skills required for appropriate patient care [7].

Alternatively, explicit assessments assign emphasis to the learning outcomes of interest and highlight the important outcomes students are required to learn and demonstrate [68]. For this reason, explicit assessments are recommended to be included in the curricula [68, 69]. The greater use of implicit over explicit assessments of cultural responsiveness in entry-level physiotherapy curricula found in this study may be due to limited research on valid and reliable methods to assess cultural responsiveness, especially in physiotherapy. That is, it may be easier to assess broad skills, such as communication or interpersonal skills if there is little guidance on how to appropriately and explicitly assess cultural responsiveness. Therefore, future research is needed to understand how assessment methods can be designed and implemented to validly and reliably assess cultural responsiveness in students. These assessment methods can then be used to effectively support students in developing safe, respectful and appropriate interactions with their patients.

How cultural responsiveness was presented and addressed in the curriculum appeared to depend on the availability of expertise, and knowledge, understanding or interest about the concept of cultural responsiveness among academic staff (Table 4). These issues were raised as challenges by the majority of participants, and have also been reported as inhibitors to the successful integration of cultural responsiveness in health education curricula [70, 71]. Without understanding, being aware, or having an interest in how culture impacts physiotherapy practice, or how interventions can be culturally adapted, academic staff are likely to struggle to facilitate the development of cultural responsiveness in students. Additionally, investment and commitment from staff are considered crucial for creating an environment that supports and advocates culturally responsive practice [58]. Students need explicit ‘role models’ who send clear messages about the importance of incorporating cultural factors into practice and about the moral obligation of interacting with patients safely and respectfully, promoting human rights and tackling health inequities [58, 72]. Therefore, future directions may include professional development to support academic staff in fostering cultural responsiveness and to build greater staff awareness, understanding, and commitment in this area.

The challenges of embedding cultural responsiveness in physiotherapy education are understandable as there is a lack of published clinical research outlining culturally responsive best practice interventions for indigenous and CALD populations [73]. While there are tools available in the literature which guide curricula content, such as the Tool for Assessing Cultural Competence Training [74], without evidence to demonstrate how physiotherapy practice can be culturally adapted for culturally diverse populations, designing curricula and understanding how cultural responsiveness should be taught and assessed remains problematic for academic staff [73]. Future research outlining what culturally responsive physiotherapy practice encompasses, and how evidence-based treatments can be adapted to improve patient outcomes should be considered to guide teaching and practice if the profession is committed to reducing health inequities. Not doing so may result in failure to take proper care of people from culturally diverse communities. Additionally, incorporating community partnerships such as engaging with indigenous and CALD community groups in curriculum development and instruction, and in assessing the appropriateness of physiotherapy treatments is another strategy that can be adopted to guide teaching and practice.

This study had a response rate of 86% and data was collected from 82% (NZ = 2 (100%), Australia = 22 (81%)) of all physiotherapy programs. The sample was also broad, including undergraduate, graduate entry masters and extended masters programs at public and private universities located in diverse geographical areas. Thus, the sample was representative of the physiotherapy program designs in 2017. However, the findings of this study also need to be considered in light of the following limitations. The results of this study were based on participants’ perceptions of their curriculum. There may have been an underestimation or overestimation of the educational content and approaches integrated, especially if participants were not involved directly with delivering material related to culture or cultural responsiveness in the classroom or clinical setting. Also, the structure and type of questions may have limited the degree to which participants provided an in-depth response about the extent or how content related to culture or cultural responsiveness was addressed in the curriculum. Additionally, while the focus group discussion only consisted of academics in one country, the focus group discussion and the questions for the interview guide were informed by the international literature on cultural responsiveness across different health disciplines, which should have captured all key elements. Finally, differences in curricula associated with the content and teaching related to Māori and Aboriginal and Torres Strait Islander populations could exist between NZ and Australia given the socio-political circumstances of each country. In NZ, Māori are acknowledged specifically in their own right as tangata whenua in a nationally diverse population [75]. Whereas in Australia, Aboriginal and Torres Strait Islander peoples do not have an officially acknowledged status [76]. This study did not aim to explore culturally responsive curricula solely from a Māori or Aboriginal and Torres Strait Islander perspective, and as such, did not did examine differences between countries. The remarkable social, political and historical contexts surrounding indigenous peoples in Australia and NZ require specific attention. In Australia and NZ, there is a moral obligation to ensure that students and health professionals learn about, understand and respect the history and traditions of indigenous peoples [13, 75]. This is essential to support reconciliation and to foster culturally responsive practices by ensuring that health professionals understand and respond to how colonialism creates and sustains health inequities [77]. Therefore, studies specifically exploring Māori and Aboriginal and Torres Strait Islander content related to cultural responsiveness in physiotherapy would be beneficial to evaluate curricula and areas for improvement to tackle health inequities that persist in these communities.

## Conclusion

Health professionals have an ethical and moral responsibility to provide healthcare that supports culturally diverse communities to achieve their maximum health and wellbeing. Appropriate education during the pre-professional years is considered important to develop the foundations needed to support the health of people from these communities. The present study offers the first insight into the learning and teaching of content related to culture and cultural responsiveness in entry-level physiotherapy programs in Australia and NZ. The results of this study demonstrate the integration of educational content and approaches related to culture or cultural responsiveness across all programs, but also highlight potential areas for further evaluation and improvement. These results lay a foundation for how learning and teaching to foster cultural responsiveness among physiotherapy students in Australia and NZ can be improved, with future research evaluating the impact of learning activities, assessments on both development of cultural responsiveness and the subsequent impact on patient outcomes. Finally, while this study is specific to Australia and NZ, these findings may offer insights about the learning and teaching considerations that may be transferrable to physiotherapy education in other countries and health disciplines.

## Additional file

Additional file 1: Final Interview guide. (DOCX 19 kb)

## Data Availability

The datasets used and/or analysed during the current study are available from the corresponding author on reasonable request.

## Abbreviations

CALD: Culturally and linguistically diverse
NZ: Aotearoa New Zealand
