# Supplementary material for: Fostering cultural responsiveness in physiotherapy: curricula survey of Australian and Aotearoa New Zealand physiotherapy programs
Source: BMC Med Educ. 2019 Aug 30;19:326. doi: 10.1186/s12909-019-1766-9 (PMC6717323; doi:10.1186/s12909-019-1766-9)
Supplement: Supplementary file 1 — Final Interview guide. (DOCX 19 kb) [file 12909_2019_1766_MOESM1_ESM.docx]

**Additional file 1: Final Interview Schedule**

**Curriculum Survey – Fostering culturally responsive practices**

Cultural responsiveness describes the ability of health professionals to respond to the healthcare needs of people from culturally diverse backgrounds. This involves being respectful, being able to communicate, understand and empathise with how people from culturally diverse backgrounds might perceive, think, behave and make judgements about their health, and also being able to adapt health practices to meet the needs of people from culturally diverse backgrounds.

This survey seeks to understand how learning and teaching is delivered in entry-level physiotherapy programs in Australia and Aotearoa New Zealand, that aims to ensure graduates are culturally responsive in their practices. Specifically, we are interested in the educational content and processes related to culture and/or cultural responsiveness, that is embedded in the curriculum to foster culturally responsive practices.

1. **Which entry level physiotherapy programs are available at your University?** Please tick all that apply.

- Undergraduate
- Graduate Entry Masters (GEM)
- Masters extended/doctorate

The following questions relate to the overall program and how learning and teaching relating to culture, cultural diversity and/or cultural responsiveness is integrated into the curriculum.

1. **How is the content related to culture and/or cultural responsiveness structured in the physiotherapy program?**

Please tick all that apply. If there is more than one program at your university (e.g. undergrad and GEM), please answer separately for each program. You can specify the programs in the columns on the right side of the table.

|  | **Program** | |
| --- | --- | --- |
|  | <specify program here> | <specify program here> |
| Addressed as an identifiable, stand-alone unit/subject in the curriculum |  |  |
| Integrated across the curriculum in a number of units/subjects |  |  |
| Not part of the curriculum *(skip to question 10)* |  |  |
| **Other (please explain):** | | |

1. **During which year(s) is the content related culture and/or cultural responsiveness mainly addressed in the physiotherapy program?**

Please tick all that apply.

| **Undergrad** | - 1^st^ year | - 2^nd^ Year | - 3^rd^ Year | - 4^th^ Year |
| --- | --- | --- | --- | --- |
| **GEM** | - 1^st^ Year | - 2^nd^ Year |  |  |
| **Masters extended** | - 1^st^ Year | - 2^nd^ Year | - 3^rd^ Year |  |
| - Other, please specify ……………………………………………………………………………………………………………………………………………………………………………………………………………………………………………………………… | | | | |

1. **What are the names of the units/subjects that include content related to culture and/or cultural responsiveness in the physiotherapy program(s)?**

Could you also indicate which program and year it is taught, and whether this is a stand-alone unit/subject or whether culture, cultural diversity and/or cultural responsiveness is integrated into the unit/subject.

| **Unit/subject name** | **Program** | **Year taught** | **Stand-alone** | **Embedded** |
| --- | --- | --- | --- | --- |
| E.g. Culture 101 | Undergrad | 2^nd^ | x |  |
|  |  |  |  |  |
|  |  |  |  |  |
|  |  |  |  |  |
|  |  |  |  |  |
|  |  |  |  |  |
|  |  |  |  |  |
|  |  |  |  |  |

1. **Could you please indicate whether the following topics related to culture and/or cultural responsiveness are addressed in the units that you named earlier?**

Please tick all that apply. If there is more than one program at your university (e.g. undergrad and GEM), please answer separately for each program. You can specify the programs in the columns on the right side of the table.

| **Content /Topic** | **Program** | |
| --- | --- | --- |
|  | <specify program here> | <specify program here> |
| Definition of cultural responsiveness/competency/awareness/sensitivity |  |  |
| Definition and concept of ethnicity and culture |  |  |
| Epidemiology of healthcare disparities |  |  |
| Factors underlying healthcare disparities |  |  |
| Demographic patterns of disparities |  |  |
| Impact of stereotyping in healthcare |  |  |
| Bias, discrimination and prejudice |  |  |
| Impact of culture on health |  |  |
| Different health beliefs and attitudes towards illness |  |  |
| Aboriginal and Torres Strait Islander health and issues |  |  |
| Māori and/or Pacific Islander health and issues |  |  |
| Culturally and linguistically diverse community health and issues |  |  |
| Immigrant and refugee health and issues |  |  |
| Self-reflection about ones’ own culture, attitudes, or beliefs |  |  |
| Communication skills – relating to communicating and interacting with patients from culturally diverse backgrounds |  |  |
| Working with interpreters |  |  |
| History taking/interviewing with reference to the cultural background and health beliefs of patients |  |  |
| Negotiating and adapting interventions for culturally diverse patients |  |  |
| **Other topics not listed included in your program:** | | |

**Process of education**

1. **What processes are used to deliver the content related to culture and/or cultural responsiveness?**

Please tick all that apply.

- Lectures/seminars (by academics/staff members)
- Films/videos
- Role play/games
- Case studies/scenarios
- Small group discussions
- Guest lectures/presentations by community members
- Personal reflection journals (focusing on cultural factors)
- Online/web-based
- Clinical placements (targeting culturally diverse populations)
- Simulated patients with a cultural component and focusing on cultural learning outcomes
- Readings
- Other: …………………………………………………………………………………………

1. **In the units/subjects that you named earlier, can you indicate the assessment practices that are used to evaluate the content that aims to foster culturally responsive practice?**

Could you also indicate whether the assessment practices explicitly or implicitly assess culturally responsive practice? (please add more rows in the table below if needed)

Explicit – The assessment criteria focus on learning outcomes related to culturally responsive practice. E.g. A written case study assignment on treating patients from culturally diverse backgrounds.

Implicit – The assessment criteria do not directly focus on learning outcomes related culturally responsive practice. However, the evaluation of cultural responsiveness is implied. E.g. Students are evaluated on being able to communicate and assess patients effectively and appropriately using the Assessment of Physiotherapy Practice (APP).

| **Assessment Practice** | **Explicit** | **Implicit** |
| --- | --- | --- |
| e.g. Written case study assignment | x |  |
| e.g. Practical assessment using (APP) |  | x |
|  |  |  |
|  |  |  |
|  |  |  |
|  |  |  |

1. **What is used to inform the content and teaching to foster culturally responsive practice in the physiotherapy program(s)?**

Please tick all that apply. Please rate on a scale how valuable the items you have ticked have been with informing the content and teaching to foster culturally responsive practice?

|  | **Please tick** | **Extremely valuable** | **Moderately valuable** | **Minimally valuable** |
| --- | --- | --- | --- | --- |
| Clinical guidelines |  |  |  |  |
| Evidence based curricula guidelines or frameworks |  |  |  |  |
| National policy frameworks |  |  |  |  |
| Theoretical models |  |  |  |  |
| Physiotherapy practice thresholds in Australia and Aotearoa New Zealand |  |  |  |  |
| Accreditation standards for Physiotherapy practitioner programs |  |  |  |  |
| **Other (Please specify):** |  |  |  |  |

1. **What are the reasons for including content that aims to foster culturally responsive practice in the physiotherapy program(s)?**

Please tick all that apply.

- Program accreditation requirements
- Clinical practice requirements
- Demographics – a diverse population
- Leadership commitment
- Faculty expertise/interest
- Other (please specify): …………………………………………………………………………………………

1. **What are the challenges associated with including and delivering content to foster culturally responsive practice in the physiotherapy program?**

.......................................................................................................................................................................................................................................................................................................................................................................................................................................................................................................................................................................................................................................................................................................................................................................................................................................................................................................................................

1. **Overall, do you think the current curriculum is effective in fostering the development of cultural responsiveness in physiotherapy students and graduates?** (Why?/ Why not?)

.......................................................................................................................................................................................................................................................................................................................................................................................................................................................................................................................................................................................................................................................................................................................................................................................................................................................................................................................................

**Thank you for completing the survey.**
